# A Novel Extraction Procedure of Contact Characteristic Parameters from Current–Voltage Curves in CdZnTe and CdTe Detectors

**DOI:** 10.3390/s23136075

**Published:** 2023-07-01

**Authors:** Fabio Principato, Manuele Bettelli, Andrea Zappettini, Leonardo Abbene

**Affiliations:** 1 Department of Physics and Chemistry (DiFC)-Emilio Segrè, University of Palermo, Viale delle Scienze, Edificio 18, 90128 Palermo, Italy; fabio.principato@unipa.it; 2 IMEM/CNR, Parco Area delle Scienze 37/A, 43100 Parma, Italy; manuele.bettelli@imem.cnr.it (M.B.); andrea.zappettini@imem.cnr.it (A.Z.)

**Keywords:** CdZnTe detectors, CdTe detectors, semiconductor–metal interface, current–voltage characteristics, interfacial layer–thermionic–diffusion theory, barrier height

## Abstract

The estimation of the characteristic parameters of the electrical contacts in CdZnTe and CdTe detectors is related to the identification of the main transport mechanisms dominating the currents. These investigations are typically approached by modelling the current–voltage *(I*–*V*) curves with the interfacial layer–thermionic-diffusion (ITD) theory, which incorporates the thermionic emission, diffusion and interfacial layer theories into a single theory. The implementation of the ITD model in measured *I*–*V* curves is a critical procedure, requiring dedicated simplifications, several best fitting parameters and the identification of the voltage range where each transport mechanism dominates. In this work, we will present a novel method allowing through a simple procedure the estimation of some characteristic parameters of the metal–semiconductor interface in CdZnTe and CdTe detectors. The barrier height and the effects of the interfacial layer will be evaluated through the application of a new function related to the differentiation of the experimental *I*–*V* curves.

## 1. Introduction

Currently, cadmium telluride (CdTe) and cadmium zinc telluride (CZT or CdZnTe) detectors represent very appealing products for X-ray and gamma ray detection [1,2,3,4], allowing sub-keV energy resolution near room-temperature conditions [5,6,7,8]. Low leakage currents (<1 nA) from the detectors are mandatory for excellent room temperature performance and optimal matching with low noise preamplifiers [5]. Generally, the leakage currents are controlled by both the bulk resistivity of the material and the electrical contacts [9,10,11,12,13]; the bulk resistivity dominates the currents at low voltages, the electrical contacts at high voltages. Typically, high-resolution CZT detectors are fabricated with quasi-ohmic electroless contacts (platinum, gold electrodes) [9,13,14,15,16], showing symmetric current–voltage (*I*–*V*) curves. CdTe detectors are equipped with rectifying contacts (indium [17], aluminium [18,19]), characterized by asymmetric *I*–*V* curves and very low currents at reverse bias; despite this, temporal instabilities, due to bias-induced polarization phenomena [2,8,19,20,21,22], must be taken into account, especially at high temperatures. Recently, very low leakage currents were obtained in new high-flux HF-CZT detectors designed for high flux measurements; these detectors are equipped with sputtered platinum contacts, showing asymmetric *I*–*V* curves and no temporal instabilities [7,23,24,25,26,27,28].

The analysis of the *I*–*V* characteristics under dark conditions is a consolidated technique to characterize the quality of the electrical contacts. This technique allows for the identification of the main transport mechanisms dominating the currents and the estimation of some characteristic parameters of the metal–semiconductor junction. The knowledge of these parameters can be useful to improve the quality of the detector metal contacts. The currents are dominated by different carrier transport mechanisms depending on the voltage range. At high reverse-bias voltages, the leakage current is characterized by the Schottky barrier of the metal–semiconductor junction. The transport mechanisms of the electrical contacts of CdTe and CZT detectors are typically investigated by using the interfacial layer–thermionic-diffusion (ITD) theory [29,30]. This model combines the thermionic emission, diffusion and interfacial layer theories into a single theory. The model adds the effects of an insulating oxide layer between the metal and the semiconductor material, generally formed during the contact deposition. The ITD modelling was successfully applied in the analysis of the *I*–*V* curves of several CZT detectors (Pt/CZT/Pt [29,31], Au/CZT/Au [9,13,15,32]).

The implementation of the ITD model in the measured *I*–*V* curves is a complex procedure, mainly due to the difficulties in correctly identifying the voltage range where each transport mechanism dominates; moreover, the modelling often requires custom simplifications and a high number of characteristic parameters, leading to overfitting and cumbersome procedures.

In this work, we will present a simple and useful method allowing for the estimation of some characteristic parameters of the metal–semiconductor interface in CZT and CdTe detectors. The method is based on the use of a new function related to the differentiation of the *I*–*V* curves. After a quick introduction t the ITD theory, we will describe the main characteristics of the new function (*H function*) applied to calculated *I*–*V* curves following the ITD model. The results from the *H function* method applied to the experimental *I*–*V* curves of different CZT and CdTe detectors will be shown.

## 2. Overview of the Interfacial Layer–Thermionic-Diffusion (ITD) Theory

The modelling of the *I*–*V* curves of CZT and CdTe detectors, the identification of the main transport mechanisms and the estimation of the characteristic parameters of the electrical contacts are typically performed by using the ITD theory [29,30]. This model, beside the thermionic-diffusion mechanism, includes the presence of an interfacial layer and its voltage drop, which can lead to a lowering of the barrier height. Typically, the detectors are modelled as metal–semiconductor–metal (MSM) systems, with two back-to-back Schottky barriers [29]. At high voltages, where the detectors are fully depleted, the transport mechanism is dominated by one of the two junctions, with a current density *J* expressed as follows [30]:

(1) $$J=\frac{A^{*}\cdot \vartheta }{1+\frac{\vartheta \cdot V_{R}}{V_{D}}}\cdot e^{-\frac{\varphi_{B0}}{V_{TH}}}\cdot e^{\frac{C_{2}V}{V_{TH}}}$$

where:

- *θ* with 0 ≤ *θ* ≤ 1 is the transmission coefficient across the interfacial layer (*θ_n_* for majority carriers represented by electrons, *θ_h_* for holes); *θ* is related to the thickness of the interfacial layer and the effective barrier height presented by the thin interfacial layer;
- *ϕ*_*B*0_ is the barrier height under thermal equilibrium conditions of the metal–semiconductor junction;
- A* is the is the effective Richardson constant of the majority charge carriers;
- *V_TH_ = k T/q*, where *k* is the Boltzmann constant, *T* the absolute temperature and *q* the electron charge;
- V_R_ = A* *T*^2^*/qN_x_* is the thermal velocity in the current flow direction; *N_x_* is the effective density of states (*x* = *v* for valence and *x* = *c* for conduction band);
- *V_D_* is the effective diffusion velocity, associated with the transport of the majority carriers from the edge of the depletion layer to the potential peak;
- *C*_2_*= ϵ_i_/(ϵ_i_ + q*^2^*Ds δ),* where *ϵ_i_* = *ϵ_r_ ϵ*_0_ and *δ* are the permittivity and thickness of the interfacial layer and *D_s_* is the density of surface states per unit energy and area; this parameter characterizes the barrier lowering due to the voltage drop across the interfacial layer.

**Figure 1:**
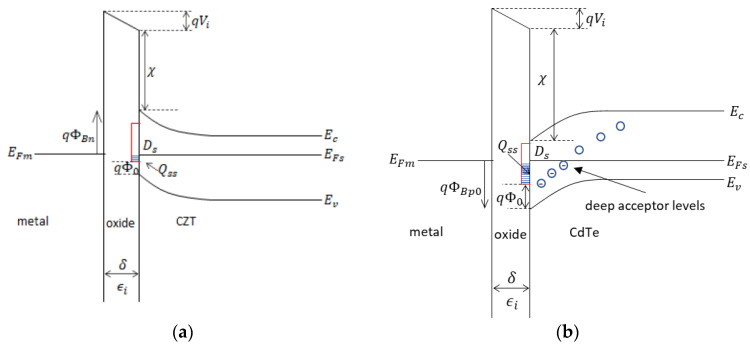
Schematic energy band diagram at the thermal equilibrium of the n-type CZT (**a**) and (**b**) p-type CdTe contacts, with an interfacial layer of the order of atomic distance. Uniform distribution of acceptor-type surface states with density *D_s_*. The surface state charge density is *Qss =* −*q D_s_ (E_g_* − *qΦ_0_)*. For the CdTe are indicated the deep acceptor levels responsible for polarization. The image force-induced lowering is neglected.

A critical issue in modelling the measured *I*–*V* curves with Equation (1) is represented by the relation between *V_D_* and the bias voltage. In Ref. [30], the analytical expression for *V_D_* is reported for the electrons, which includes the voltage drop across the interfacial layer and the transmission coefficient of carriers across the interfacial layer. This expression involves the electrostatic potential profile in the depleted region. In Ref. [29], a simplified expression for *V_D_* is used, as follows:

V_D_ = µ_n_ E_c_, (2)

where *µ_n_* is the electron mobility and *E_c_* is the electric field at the cathode, which is assumed to depend linearly on the reverse bias voltage. The parameter *C*_2_ characterizes the voltage drop across the interfacial layer, *C*_2_ *V*, which gives the additional barrier height lowering. *C_2_* is assumed to be independent by the temperature [29]. When the density of surface states *D_s_* → *∞*, then *C*_2_ → 1, and in this case the Fermi level at the interface is pinned at a value Φ_0_ above the valence band by the surface states and the barrier height seen by electrons is *ϕ_Bn_ = E_g_* − *Φ*_0_, where *E_g_* is the band gap of the semiconductor. In this case, the barrier height is independent of the metal work function. When *D_s_* → 0, *ϕ_Bn_ = φ_m_* − *χ*, where *φ_m_* is the metal work function and χ the electron affinity of the semiconductor. Typical *C*_2_ values in CZT detectors are of the order of 10^−5^ and 10^−4^ [15,29,31,32]. Figure 1 shows the simplified schematic diagrams of the metal/n-CZT (a) and metal/p-CdTe (b) interface, at thermodynamic equilibrium.

The ITD theory foresees two extreme cases:

(i) If *θ V_R_ << V_D_*, the leakage current is dominated by the thermionic emission (TE) and Equation (1) can be approximated to:
  (3) $$J=A^{*}\cdot \vartheta \cdot T^{2}\cdot e^{-\frac{\varphi_{B0}}{V_{TH}}}\cdot e^{\frac{C_{2}V}{V_{TH}}}$$
(ii) If *θ V_R_ >> V_D_*, the leakage current is dominated by the diffusion (D) mechanism and can be presented as:
  (4) $$J=qN_{x}V_{D}\cdot e^{-\frac{\varphi_{B0}}{V_{TH}}}\cdot e^{\frac{C_{2}V}{V_{TH}}}$$

If the interfacial layer is not present (i.e., *θ =* 1 and *C*_2_ *=* 0) or its effect is negligible, the leakage current is diffusion-limited to high-bias voltages that show quasi-ohmic behaviour, with effective resistivity higher than the bulk one [29].

## 3. Estimation of the Characteristic Parameters of Electrical Contacts: The Role of the H Function

The ITD modelling of the measured *I*–*V* curves of CZT and CdTe detectors is very helpful for the estimation of the characteristic parameters of the electrical contacts. However, the implementation of the ITD model often results in a complex fitting procedure, characterized by several free parameters, requiring dedicated simplifications and the selection of the proper voltage/temperature range for each dominant mechanism. In order to simplify the parameter estimation, we defined a new function, termed the *H* function, expressed as follows:

(5) $$H(V,T)=\frac{V_{TH}}{J}\frac{\partial J}{\partial V}$$

By applying the *H* function on the *I*–*V* curves modelled with the ITD theory (Equation (1)), we obtain:

(6) $$H\left(V,T\right)=\frac{V_{TH}}{V_{D}}\frac{\partial V_{D}}{\partial V}\frac{1}{1+\frac{V_{D}}{\vartheta V_{R}}}+C_{2}$$

If the TE mechanism dominates the current, the *H* function can be approximated as follows:

*H (V,T) = C*_2_ (7)

This result highlights that the presence of the plateau zone on the *H* function can allow for the simple and well-defined identification of the TE voltage range. Therefore, the behaviour of the *H*–*V* curves can be used to estimate the *C_2_* parameter and identify the *I*–*V* zone dominated by the TE mechanism. The knowledge of both *C_2_* and the current values in the TE regime is key in the estimation of further characteristic parameters of the electrical contacts. The slope and the intercept of the linear Arrhenius plots of ln(J/T^2^) − C_2_V/V_TH_ versus *q/KT* (from Equation (3)) give the estimation of the barrier height *φ_B0_* and the product A*θ. Hence, the *H* function allows us to determine the TE voltage range and estimation of the characteristic parameters by taking into account the barrier lowering (through the parameter *C_2_*).

To better highlight the potentialities of the *H* function, we calculated some *I*–*V* curves expected from the ITD theory (Equation (1)). We used the characteristic parameters of Pt/CZT/Pt detectors [29], as reported in Table 1.

**Figure 2:**
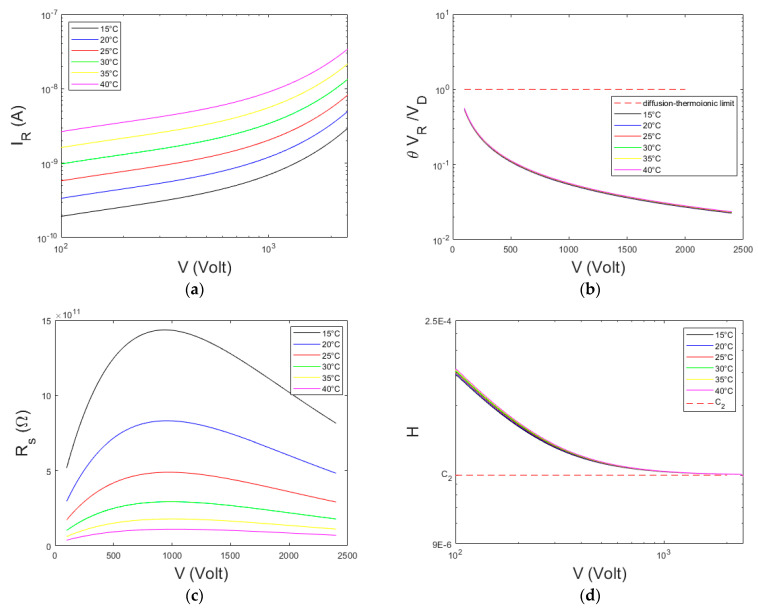
(**a**) Calculated *I*−*V* curves following the ITD model (Equation (1)) for a Pt/CZT/Pt detector (Table 1). (**b**) The ratio *θ V_R_/V_D_* vs. the voltage, (**c**) The *R_s_*−*V* curves. (**d**) The calculated *H*−*V* curves. The results are presented at different temperatures and by using a transmission coefficient *θ =* 0.05.

The ratio *θ V_R_/V_D_*, the resistance *R_S_ = V/I* and the *H* function vs. the voltage were calculated at different temperatures. The calculated *I*–*V* curves, presented in Figure 2a, highlight a linear trend up to about 1000 V, followed by an exponential trend. The ratio *θ V_R_/V_D_* is always <1 and it decreases at high voltages (Figure 2b). This indicates that the currents are dominated by the TE mechanism. The *R_s_*–*V* curves (Figure 2c) present a maximum value at the voltage *V_max_ = V_TH_/C*_2_, as expected from the TE regime. This allows another possible approach in *C_2_* estimation. The *H*–*V* curves (Figure 2d) clearly show the expected plateau zone, giving a *C_2_* value equal to the settled one (Table 1, *C*_2_ = 2.5·10^−5^). Both the ratio *θ V_R_/V_D_* and the *H* function are quite independent from the temperature. We also estimated *C*_2_ from the *R_s_*–*V* curves, obtaining *C*_2_ = 2.6·10^−5^.

**Figure 3:**
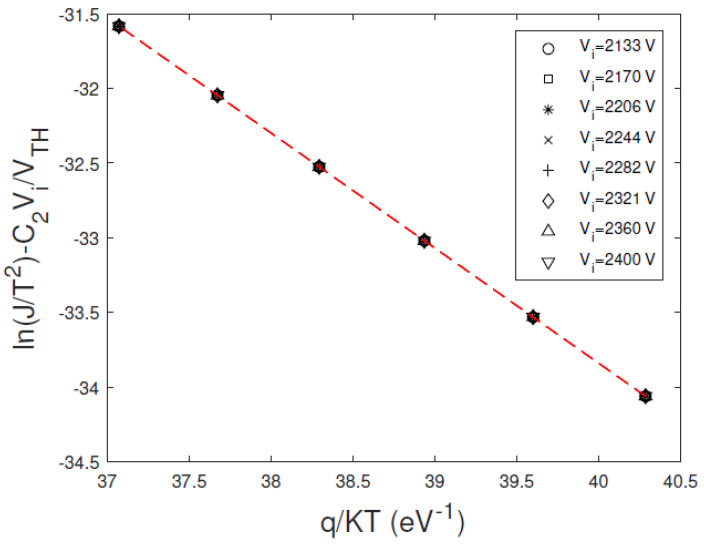
Calculated Arrhenius plots of ln(J/T^2^) − C_2_V/V_TH_ versus *q/KT*, at different bias voltages. The voltages belong to the voltage range where the *H*−*V* curve reaches a plateau (dominant TE regime). We used a transmission coefficient *θ =* 0.05.

This small discrepancy is due to the *V_max_* value, positioned in a voltage zone where the dominance of the mechanism is not marked. Figure 3 shows the Arrhenius plots of ln(J/T^2^) − C_2_V/V_TH_ versus *q/KT*, calculated at different bias voltages, where TE dominates. The curves are independent of the bias voltage, giving through the slope and the intercept a correct estimation of the barrier height *qφ_B_*_0_ and the transmission coefficient *θ*, in perfect agreement with the settled data of Table 1.

**Figure 4:**
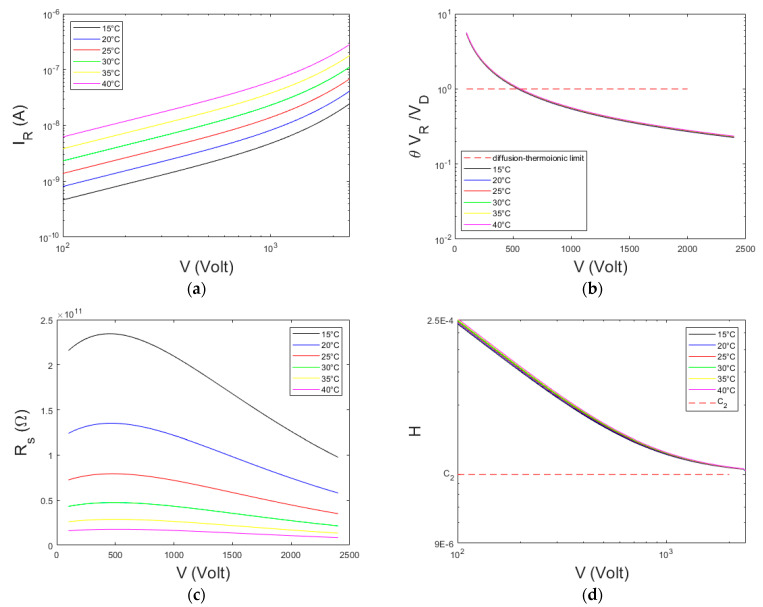
(**a**) Calculated *I*–*V* curves following the ITD model (Equation (1)) for a Pt/CZT/Pt detector (Table 1). (**b**) The ratio *θ V_R_/V_D_* vs. the voltage, (**c**) The *R_s_*–*V* curves. (**d**) The calculated *H*–*V* curves. The results are presented at different temperatures and by using a transmission coefficient *θ =* 0.5.

As comparison, we also calculated the *I*–*V* curves and related quantities by using a greater transmission coefficient *θ =* 0.5. In this case, the diffusion component gives higher contribution to calculated currents. The results are shown in Figure 4. The *H* function does not reach a well-defined plateau (Figure 4d), obtaining *C*_2_ = 2.7·10^−5^. The maximum of the *R_s_* curves occur at low voltages, obtaining *C*_2_ = 5.4·10^−5^, very different from the settled value (*C*_2_ = 2.5·10^−5^).

**Figure 5:**
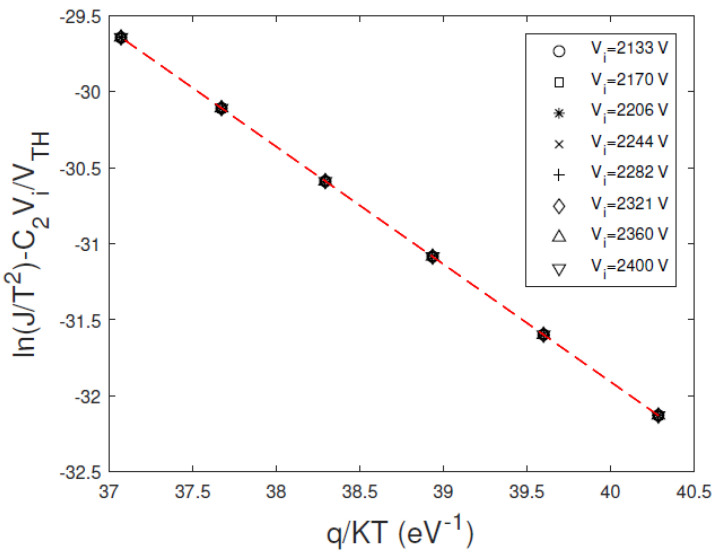
Calculated Arrhenius plots of ln(J/T^2^) − C_2_V/V_TH_ versus *q/KT*, at different bias voltages. The voltages belong to the voltage range where the *H*–*V* curve reaches a quasi-plateau. We used a transmission coefficient *θ =* 0.5.

From the Arrhenius plots of Figure 5 we obtained *qφ_B_*_0_ in perfect agreement with the settled data of Table 1, while a transmission coefficient *θ* = 0.35 was estimated.

Generally, the estimation of the *C*_2_ parameter is more accurate for the *H* function approach, and the differences between the two methods are more marked when the diffusion contribution increases.

## 4. Materials and Methods

We applied the proposed *H function* method to measured *I*–*V* curves of different CZT and CdTe detectors. The first sample (*D*1 *detector*) is based on a low flux LF-CZT crystal (4.1 × 4.1 × 3 mm^3^), grown using the traveling heater method (THM) technique (Redlen Technologies, Saanichton BC, Canada); it was fabricated at the IMEM-CNR Institute (Parma, Italy) by using gold electroless contacts. Recently, very-low-noise gold contacts were realized on CZT detectors by our group [14,15,23,25,26,33], ensuring low leakage currents at room temperature (4.7 nA cm^−2^ at 1000 V cm^−1^) and good room-temperature operation, even at high bias voltages (>5000 V cm^−1^). The LF-CZT crystals are characterized by mobility–lifetime products *μ_e_τ_e_* ranging from 1 to 3·10^−2^ cm^2^/V and *μ_h_τ_h_* from 2 to 3·10^−5^ cm^2^/V [21,32,33], mainly used for electron-sensing detectors working at low flux conditions [14,34,35,36,37,38]. The anode layout is characterized by a central pixel (2 × 2 mm^2^) surrounded by a guard ring. The width of the guard ring is 950 μm, and the gap between the pixel and the guard ring is 50 μm. The cathode is a planar electrode covering the overall detector surface (4.1 × 4.1 mm^2^).

The second sample (*D*2 *detector*) was also realized at the IMEM-CNR Institute; it is based on a high-flux HF-CZT crystal (5 × 5 × 1.5 mm^3^) with sputtered platinum (Pt) electrical contacts. Recently, HF-CZT crystals grown using the THM technique are developed by Redlen. These crystals, characterized by enhanced hole charge transport properties (*μ_e_τ_e_* ranging from 2 to 3·10^−3^ cm^2^/V and *μ_h_τ_h_* from 1 to 2·10^−4^ cm^2^/V [23]), are very appealing for high flux measurements [23,24,25,26,27,28]. The detector has a full-area cathode with the Pt contact and a customized pixelated Pt anode (2 × 2 array; pixel size 500 × 500 μm^2^, with 200 μm gap). The pixels are surrounded by a guard ring.

The third sample (*D*3 *detector*) is based on a CdTe crystal (4.1 × 4.1 × 2 mm^3^), manufactured by Acrorad (Japan). The anode and cathode geometries are identical to the D1 detector ones. The detector is characterized by the Al/CdTe/Pt electrode configuration. High-resolution performances were generally obtained with Al/CdTe/Pt detectors at low X-ray energies (<100 keV) by using moderate cooling and taking into account polarization effects [2,8,19,20,21,39,40,41].

The main characteristics of the detectors are summarized in Table 2.

The *I*–*V* curves of these detectors were measured with the Keithley 2410 and CAEN NDT1471 instruments, providing the cathode bias voltage. The Keithley 2635B, configured as an electrometer and connected to the pixel anode, was used to measure the leakage current (accuracy < 0.2%). The guard-ring electrodes are forced to the ground potential. *I*–*V* measurements were performed in both reverse (i.e., by applying negative voltages to the full-area electrodes) and in forward biasing. All measurements were performed with the detectors enclosed in a shielded box under a nitrogen atmosphere with a temperature control system. To minimize the polarization effects in the CdTe detector (D3 detector), a dedicated procedure was used by resetting the bias voltage between two consecutive measurements.

## 5. Measurements and Results

### 5.1. Experimental Current-Voltage (I–V) Curves

**Figure 6:**
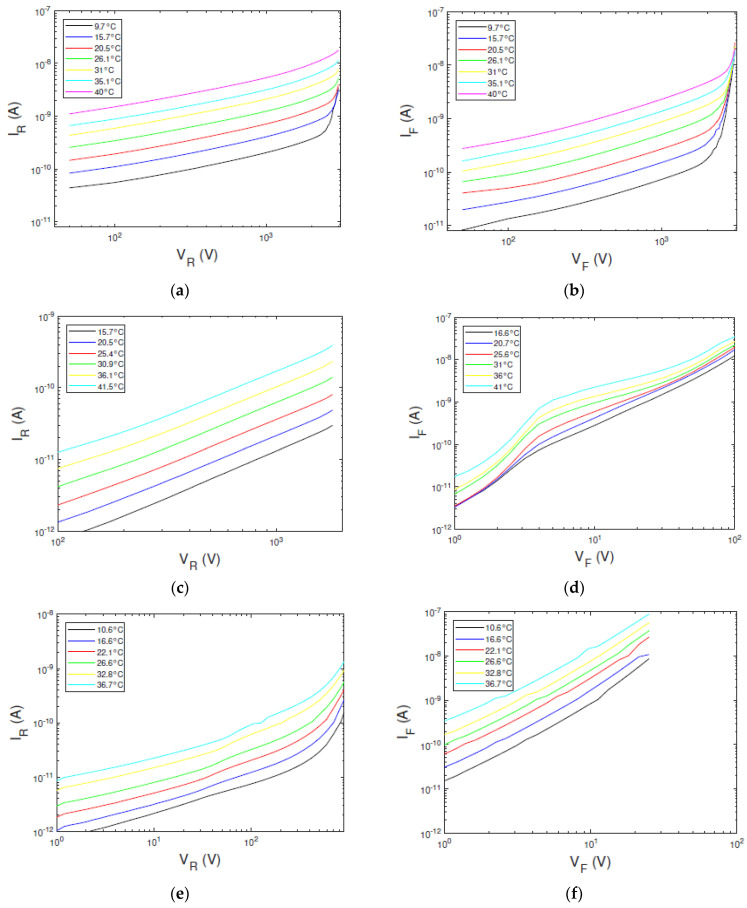
Measured *I*−*V* curves at different temperatures for the three types of detectors. The left column and the right column show the reverse and forward biasing currents, respectively. (**a**,**b**) The *I*−*V* curves of D1 detector, (**c**,**d**) D2 detector, and (**e**,**f**) D3 detector.

Figure 6 shows the *I*–*V* curves of the investigated detectors at different temperatures, measured in reverse (left column) and forward (right column) biasing. The D1 detector (Au/LF-CZT/Au, electroless deposition) is characterized by quasi-symmetric current curves, typically observed in Au/CZT/Au detectors with electroless contacts [15,32]. At low voltages, the current follows a power law trend *∝ V^b^*, with *b =* 0.8 in reverse (Figure 6a) and *b =* 0.9 in forward (Figure 6b). At high voltages the current increases with a higher slope (*b* > 2), both in reverse and forward, typical of the space charge limited current (SCLC) regime [32]. The starting voltage of the SCLC regime depends on the temperature. The D2 detector (Pt/HF-CZT/Pt, sputter deposition) shows asymmetric *I*–*V* curves, typical of CZT detectors with sputter Pt contacts [7], with forward-biasing currents (Figure 6d) higher than the reverse-biasing ones (Figure 6c). The transport mechanism in reverse is governed by a reversed-biased Schottky barrier, with the ITD model able to be applied. The reverse current follows a trend ∝ V at all temperatures, typical of the diffusion mechanism. Asymmetric *I*–*V* curves were also measured for the D3 detector (Al/CdTe/Pt), in agreement with the well-known rectifying properties of Al/CdTe/Pt detectors [18,19,41]. At reverse voltages less than 100 V (Figure 6e), the currents follow the trend ∝ V^1.2^; at higher voltages, the *I*–*V* curves are characterized by exponential behaviour due to the barrier-lowering effects. The forward currents (Figure 6f) are higher than the reverse ones, showing the typical trend of forward-biased diodes with an ideality factor depending on the voltage [21]. We applied our approach to the reverse currents for all detectors; the forward currents of the D1 detector will be only analyzed.

### 5.2. Experimental Resistance-Voltage (R_s_–V) Curves

**Figure 7:**
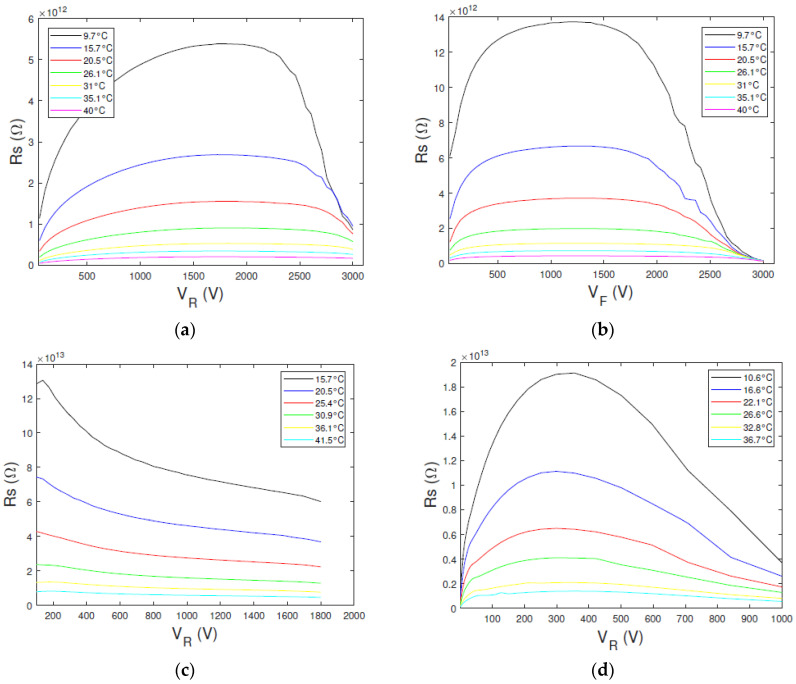
Experimental resistance-voltage *(R_s_*–*V)* curves at different temperatures for the three types of detectors. The *R_s_*–*V* curves of D1 detector obtained from the measured (**a**) reverse and (**b**) forward currents; (**c**) D2 detector and (**d**) D3 detector.

In Figure 7 are shown the resistance *R_s_* values vs. the bias voltage at different temperatures, obtained from the measured *I*–*V* curves (Figure 6). The *R_s_* curves of D1 (Figure 7a,b) and D3 (Figure 7d) detectors show a well-defined maximum value at the expected voltage of *V_max_ = q C*_2_*/KT*, in agreement with the ITD model. Therefore, the value of C_2_ can be easily estimated. The V_max_ value, for the D2 detector, is positioned in the low voltage zone (Figure 7c) due to the prevalence of the diffusion component. Hence, the C_2_ value for the D2 detector cannot be estimated with this approach.

### 5.3. Experimental H–V Curves

**Figure 8:**
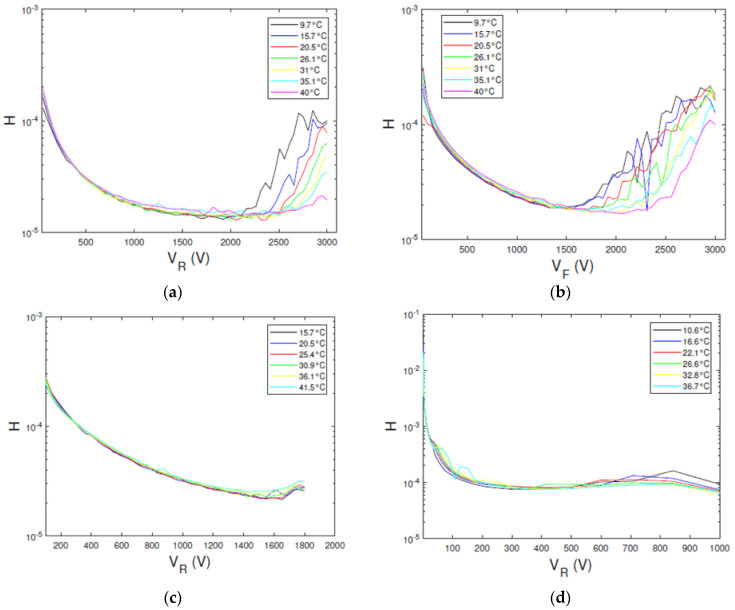
Experimental *H*−*V* curves at different temperatures for the three types of detectors. The *R_s_*−*V* curves of D1 detector obtained from measured (**a**) reverse and (**b**) forward currents; (**c**) D2 detector and (**d**) D3 detector.

The experimental *H* function values versus the bias voltage are shown in Figure 8. Generally, the *H*–*V* curves follow the expected behaviour from the ITD model. The curves are quite independent on the temperature, showing decreasing values with voltage and reaching a plateau at high voltages. The *H* function of the D1 detector reaches a plateau between 1500–2000 V and 1400–1600 V in reverse and forward biasing, respectively. The ITD model fails at high voltages, where a marked dependence on the temperature is observed (SCLC regime). A plateau is also obtained for D2 and D3 detectors (at about 1500 V and 300 V for D2 and D3, respectively), showing a low temperature dependence.

### 5.4. Evaluation of Characteristic Parameters from Experimental R_s_–V and H–V Curves

Table 3 summarizes the estimated *C_2_* values obtained from the *R_s_*–*V* and *H*–*V* curves. Generally, a good agreement is observed between the results of the two approaches.

**Figure 9:**
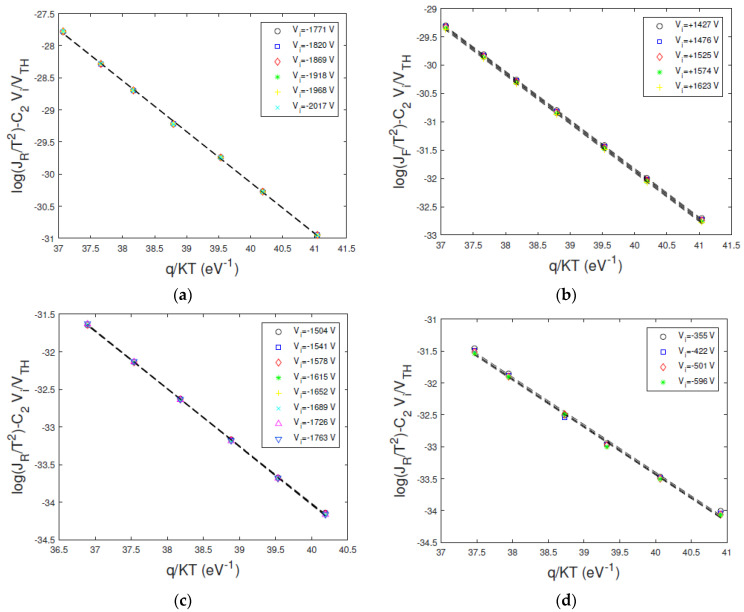
Experimental Arrhenius plots of ln(J/T^2^) − C_2_V/V_TH_ versus *q/KT* at different bias voltages. The voltages belong to the voltage range where the *H*–*V* curve reaches a plateau. The plots are calculated from the measured (**a**) reverse and (**b**) forward currents of D1 detector; (**c**) D2 detector and (**d**) D3 detector.

Figure 9 shows the Arrhenius plots of ln(J/T^2^) − C_2_V/V_TH_ versus *q/KT*, obtained at different bias voltages, properly selected in the plateau zone of the related *H*–*V* curves (Figure 8). The linear behaviour is visible, and quite good independence from the voltage is generally observed. The barrier height *φ_B_*_0_ and the transmission coefficient *θ* values were calculated from the Arrhenius plots at all voltages located in the plateau zone. For each voltage, we calculated the parameter values and their errors (95% confidence interval) though a weighted linear best fitting. In Table 4 are reported the weighted mean values of the parameters over the selected voltages.

The reverse barrier height *φ_B_*_0_ of the D1 detector is slightly lower than the forward one. This can explain the lower forward-leakage currents. The estimated value of the *θ* parameter in reverse, supposing that electrons are the majority carriers and by using a Richardson constant A* *=* 12 *(A* cm^−2^ K^−1^*)* (Table 1), is equal to *θ_n_ =* 0.36. In forward biasing, *θ_n_ ≈* 1 has no physical meaning. For the D2 detector, the value of the estimated value of the effective barrier height is 0.77 eV, which is in agreement with the literature [29,30]. We obtained a transmission coefficient *θ_n_ =* 0.048, close to the value found in [29]. By considering the holes (with A* = 87.6 (A cm^−2^ K^−1^) [42]), we estimated a *θ_h_ =* 0.0004.

The Arrhenius plots, related to the D3 detector, give a barrier height of 0.74 eV, in agreement with our previous measurements by using the resistance contact approach [21]. By considering light holes involved in the transport mechanism, a *θ_h_ =* 0.18 was estimated.

## 6. Discussion and Conclusions

We presented a novel approach to evaluate the characteristic parameters of the electrical contacts of CZT and CdTe detectors. The method is based on a simple analysis of the *I*–*V* curves through the application of a new function, termed the *H function*. The following key results are obtained:

(i) the analysis of the behaviour of *H*–*V* curves, together with the resistance–voltage curves, allows for a clear identification of the main mechanisms controlling the currents, often masked in the *I*–*V* curves;
(ii) the presence of the plateau zone in the *H*–*V* curves highlights the bias voltage range where the thermionic emission dominates; this allows for the estimation of the *C*_2_ parameter characterizing the barrier lowering due to the voltage drop across the interfacial layer;
(iii) the knowledge of both *C*_2_ and the current values in the TE regime is helpful for the estimation of further characteristic parameters of the electrical contacts. The slope and the intercept of the linear Arrhenius plots of ln(J/T^2^) − C_2_V/V_TH_ give the estimation of the barrier height *φ_B_*_0_ and the transmission coefficient *θ*. In this case, the correct identification of the TE voltage range and the introduction of the barrier lowering (through the parameter *C*_2_) allow for an accurate estimation of these parameters.

**Figure 10:**
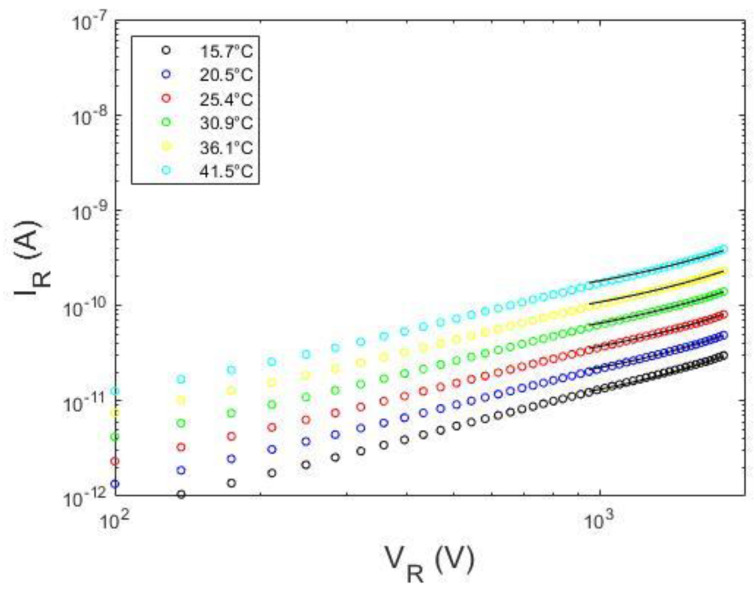
Successfully ITD modelling (Equation (1); black lines) of the measured *I*−*V* curves of the D2 detector at different temperatures.

To strengthen the results obtained from our approach, we modelled the experimental *I*–*V* curves with the ITD model (Equation (1)) by using the parameters (*θ*, *C*_2,_ *φ_B_*_0_*)* estimated with the *H function* method. The results, related to the D2 detector, are shown in Figure 10. A good agreement between the data and the ITD function was obtained. The modelling at lower voltages (<1000 V) requires further details about the expression of *V_D_* (Equation (2)).

The presence of the barrier lowering can be often masked in the *I*–*V* curves, giving non-accurate estimations of the characteristic parameters of the electrical contacts. For example, we observed a dominant diffusion mechanism in the D2 detector in the investigated temperature range. In this case, the current depends on the temperature by the following relation (Equation (4)):

(8) $$J\alpha T^{\frac{3}{2}}\cdot e^{-\frac{\varphi_{B0}}{V_{TH}}}$$

**Figure 11:**
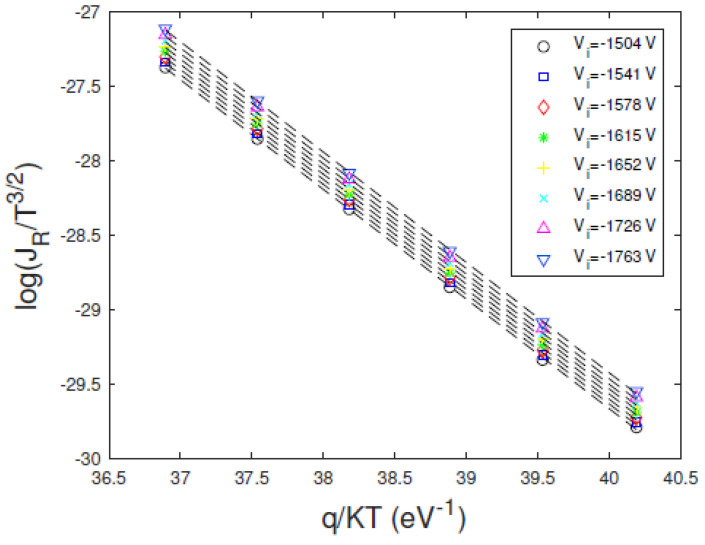
Measured Arrhenius plots of ln(J/T^3^/^2^) versus *q/KT* at different bias voltages (D2 detector). We assumed only the diffusion mechanism governing the transport mechanism. The dashed lines are the linear fits.

The barrier height is estimated by measuring the current at a range of temperatures for a fixed voltage and by fitting Equation (8) to an Arrhenius plot of the data [13]. By implementing the previous equation to the measured leakage currents of the D2 detector in the same voltage range used in the application of the *H* function method, we obtained the Arrhenius plots of Figure 11. We note that the intercept values depend on the reverse-bias voltage where the reverse current is measured, unlike the Arrhenius plots of Figure 9c. From the slopes of the linear fits of Figure 11 we obtained a *φ_B_*_0_ of 0.74 ± 0.03 eV. This underestimation of the barrier height value, if compared with the value obtained with the *H* function method, cannot be explained by the different temperature dependence of the diffusion model (i.e., *J α T* ^3/2^) with that of the thermionic emission assumed in the implementation of the *H* function (i.e., *J α T*^2^). Indeed, in the investigated temperature range the change in barrier height value due to the change in the exponent of the absolute temperature is smaller than 1%. This difference is due to the lack of the barrier-lowering term *C*_2_ *V/V_TH_* in Equation (8), which causes the underestimation of the barrier height value. We stress that when the contribution of the barrier lowering in the leakage currents is small, i.e., with *C*_2_ *V*< *V_TH_*, the expected exponential behavior can be approximated to a linear trend, therefore masking the barrier lowering.

## Figures and Tables

**Table 1:** Characteristic parameters of the electrical contacts of Pt/CZT/Pt detectors [29], used in the calculated ITD current–voltage curves. The parameters were estimated in the reverse-current regime.

*C*_2_	*qφ_B_*_0_ (eV)	A* (*A* cm^−2^ K^−2^)	*µ_n_* (cm^2^ V^−1^ s^−1^)	*θ_n_*
2.5·10^−5^	0.77	12	1000	0.05

**Table 2:** Main characteristics of the detectors used for the *I*–*V* measurements.

*Detector*	*Crystal*	*Electrodes*	*Electrode Deposition*	*Anode Pixel Size*
D1	LF-CZT 4.1 × 4.1 × 3 mm^3^	Au/CZT/Au	electroless	2 × 2 mm^2^
D2	HF-CZT 5 × 5 × 1.5 mm^3^	Pt/CZT/Pt	sputter	0.5 × 0.5 mm^2^
D3	CdTe 4.1 × 4.1 × 2 mm^3^	Al/CdTe/Pt	n.a.	2 × 2 mm^2^

**Table 3:** Estimated *C*_2_ values (95% confidence interval) from the measured *R_s_*–*V and H*–*V curves*.

	*C*_2_ (×10^−5^) *R_s_*–*V Curves*	*C*_2_ (×10^−5^) *H*–*V Curves*
Detector D1 (reverse biasing)	1.43 ± 0.15	1.43 ± 0.09
Detector D1 (forward biasing)	2.09 ± 0.14	1.88 ± 0.04
Detector D2 (reverse biasing)	n.a.	2.40 ± 0.20
Detector D3 (reverse biasing)	8.3 ± 0.4	8.4 ± 0.4

**Table 4:** Estimated *φ_B_*_0_ and *θ* values (95% confidence interval) from the experimental Arrhenius plots.

	*φ_B_*_0_ (eV)	*θ*
Detector D1 (reverse biasing)	0.792 ± 0.008	0.4 ± 0.1
Detector D1 (forward biasing)	0.859 ± 0.005	~1
Detector D2 (reverse biasing)	0.767 ± 0.004	0.048 ± 0.009
Detector D3 (reverse biasing)	0.74 ± 0.02	0.2 ± 0.1
